# Supplementary material for: Non-Alcoholic Fatty Liver Disease and Risk of Incident Type 2 Diabetes: Role of Circulating Branched-Chain Amino Acids
Source: Nutrients. 2019 Mar 26;11(3):705. doi: 10.3390/nu11030705 (PMC6471562; doi:10.3390/nu11030705)
Supplement: Supplementary file 1 [file nutrients-11-00705-s001.pdf]

Table S1. Multivariable linear regression analysis demonstrating the positive association of plasma branched-chain amino acids with an elevated hepatic steatosis index (HSI) (>36) after adjustment for clinical and laboratory covariates in 5791 subjects.

	**Model 1**		**Model 2**		**Model 3**		**Model 4**		**Model 5**	
	β	*p*	β	*p*	β	*p*	β	*p*	β	*p*
Age	0.050	<0.001	0.049	<0.001	0.047	<0.001	-0.019	0.217	-0.041	0.007
Sex (men vs. women)	0.503	<0.001	0.505	<0.001	0.502	<0.001	0.505	<0.001	0.474	<0.001
HSI >36 vs. ≤36	0.244	<0.001	0.242	<0.001	0.240	<0.001	0.231	<0.001	0.116	<0.001
Family history of T2D (yes/no)			0.044	<0.001	0.046	<0.001	0.045	<0.001	0.030	0.005
Alcohol intake (≥10 g/day)					0.018	0.107	0.021	0.060	0.022	0.046
Current smoking (yes/no)					-0.026	0.019	-0.029	0.011	-0.019	0.076
eGFR (ml/min/1.73 m^2)							-0.078	<0.001	-0.057	<0.001
UAE (mg/24 hr)							-0.007	0.557	-0.016	0.143
Use of antihypertensive medication							0.029	0.021	0.000	0.994
Use of lipid-lowering drugs							0.032	0.007	0.012	0.278
HOMA-IR									0.318	<0.001
HOMA-β									-0.045	0.083

β: standardized regression coefficients. HOMA-IR and HOMA-β were log_e transformed for analyses. BMI, body mass index; eGFR, estimated glomerular filtration rate; HOMA, Homeostasis Model Assessment; HSI, hepatic steatosis index; IR, insulin resistance; T2D, type 2 diabetes; UAE; urinary albumin excretion.

Model 1*: adjusted for age and sex.*

Model 2*: adjusted for age, sex and family history of type 2 diabetes.*

Model 3*: adjusted for age, sex, family history of type 2 diabetes, alcohol intake and current smoking.*

Model 4*: adjusted for age, sex, family history of type 2 diabetes, alcohol intake, current smoking, estimated glomerular filtration rate, urinary albumin excretion and use of antihypertensive medication and lipid-lowering drugs.*

Model 5*: adjusted for age, sex, family history of type 2 diabetes, alcohol intake, current smoking, estimated glomerular filtration rate, urinary albumin excretion, use of antihypertensive medication and lipid-lowering drugs, HOMA-IR and HOMA-β.*

Table S2. Prospective associations of HSI with incident type 2 diabetes.

	**Non-Elevated HSI** **≤36**	**Elevated HSI** **>36**	
Participants, n	4328	1463	
Incident T2D, n (%)	128 (3.0)	148 (10.1)	
	**HR (95% CI)**		***p*-Value**
Crude Model	(ref)	3.69 (2.91–-4.68)	<0.001
Model 1	(ref)	3.57 (2.81–4.52)	<0.001
Model 2	(ref)	3.47 (2.73–4.40)	<0.001
Model 3	(ref)	3.53 (2.78–4.48)	<0.001
Model 4	(ref)	3.08 (2.36–4.00)	<0.001
Model 5	(ref)	2.20 (1.62–2.99)	<0.001
Model 6	(ref)	1.99 (1.45–2.17)	<0.001

Data are presented as hazard ratio (HR) with 95% confidence interval (CI). HSI, hepatic steatosis index; BCAA, branched-chain amino acids; T2D, type 2 diabetes.

Model 1: adjusted for age and sex.
Model 2: adjusted for age, sex and family history of type 2 diabetes.
Model 3: adjusted for age, sex, family history of type 2 diabetes, alcohol intake and current smoking.
Model 4: adjusted for age, sex, family history of type 2 diabetes, alcohol intake, current smoking, eGFR, UAE, antihypertensive medication and lipid-lowering drugs.
Model 5: adjusted for age, sex, family history of type 2 diabetes, alcohol intake, current smoking, eGFR, UAE, antihypertensive medication, lipid-lowering drugs, HOMA-IR and HOMA-β.
Model 6: adjusted for age, sex, family history of type 2 diabetes, alcohol intake, current smoking, eGFR, UAE, antihypertensive medication, lipid-lowering drugs, HOMA-IR, HOMA-β and BCAAs.

Table S3. Prospective associations of BCAAs with incident type 2 diabetes.

	**BCAA per 1 SD increment**	
Participants, n	5791	
Incident T2D, n (%)	276 (4.8)	
	**HR (95%CI)**	***p*-Value**
Crude Model	1.68 (1.57–1.81)	<0.001
Model 1	1.65 (1.52–1.79)	<0.001
Model 2	1.63 (1.50–1.77)	<0.001
Model 3	1.64 (1.50–1.79)	<0.001
Model 4	1.64 (1.49–1.81)	<0.001
Model 5	1.35 (1.20–1.53)	<0.001
Model 6	1.29 (1.13–1.48)	<0.001

Data are presented as hazard ratio (HR) with 95% confidence interval (CI). HSI, hepatic steatosis index; BCAA, branched-chain amino acids; T2D, type 2 diabetes.

Model 1: adjusted for age and sex.
Model 2: adjusted for age, sex and family history of type 2 diabetes.
Model 3: adjusted for age, sex, family history of type 2 diabetes, alcohol intake and current smoking.
Model 4: adjusted for age, sex, family history of type 2 diabetes, alcohol intake, current smoking, eGFR, UAE, antihypertensive medication and-lipid lowering drugs.
Model 5: adjusted for age, sex, family history of type 2 diabetes, alcohol intake, current smoking, eGFR, UAE, antihypertensive medication, lipid-lowering drugs, HOMA-IR and HOMA-β.
Model 6: adjusted for age, sex, family history of type 2 diabetes, alcohol intake, current smoking, eGFR, UAE, antihypertensive medication, lipid-lowering drugs, HOMA-IR, HOMA-β and elevated HSI (yes/no).
